# Enrichment of Autophagy and Proteosome Pathways in Breast Muscle of Feed Efficient Pedigree Male Broilers

**DOI:** 10.3389/fphys.2018.01342

**Published:** 2018-10-26

**Authors:** Alissa Piekarski-Welsher, Elizabeth Greene, Kentu Lassiter, Byungwhi Caleb Kong, Sami Dridi, Walter Bottje

**Affiliations:** Department of Poultry Science, Center of Excellence for Poultry Science, University of Arkansas, Fayetteville, AR, United States

**Keywords:** autophagy, proteosome, feed efficiency, broiler, breast muscle

## Abstract

**Background:** Feed efficiency (FE) is an important genetic trait in poultry and livestock. Autophagy (self-eating) and proteosomes are cellular processes that remove damaged cell components (e.g., proteins, organelles). As evidence of extensive protein oxidation was observed in Pedigree Male (PedM) broilers exhibiting a low FE (LFE) phenotype compared to a high FE (HFE) phenotype, the main goal of this study was to assess gene and protein expression of the autophagy and proteosome pathways in breast muscle obtained in PedM broilers exhibiting HFE and LFE phenotypes.

**Methods:** Feed efficiency was calculated as weight gain divided by feed intake gain in individual PedM broilers that were measured between 6 and 7 weeks of age. Targeted gene expression was conducted on breast muscle using quantitative real-time polymerase chain reaction (qPCR) to determine mRNA expression of genes associated with the autophagy pathway; AMP-activated protein kinase alpha 1 (AMPKα1), mammalian target of rapamycin (mTOR), Beclin 1, and autophagy genes (Atg) 3, Atg7, and Atg16L1. Binomial distribution analysis was conducted on transcriptomic and data obtained by RNAseq and shotgun proteomics, respectively on the same set of tissues for genes associated with autophagy, vacuole formation, and proteosome expression.

**Results:** Greater efficiency was attained in the HFE PedM broilers by greater weight gain on the same amount of feed consumed resulting in FEs of 0.65 ± 0.01 and 0.46 ± 0.01 in the HFE and LFE phenotypes, respectively. Targeted mRNA expression analysis revealed significant (*P* < 0.05) elevations in AMPKa1, mTOR, Atg16L1, and Atg7 and a marginal (*P* = 0.07) elevation in Beclin1. Binomial distribution analysis transcriptomic and proteomic data revealed significant skews favoring autophagy-, vacuole-, and proteosome-related genes in the HFE phenotype. These results indicate that the autophagy and proteosome expression is enhanced in the HFE compared to the LFE pedigree male broiler phenotype suggesting that protein and organelle quality control may be enhanced in high feed efficiency.

## Introduction

De Duve and Wattiaux (1966) is credited as being the first to describe the process of autophagy (Ward et al., 2016) that plays an important role in elimination of damaged proteins and organelles (e.g., Cuervo and Dice, 2000; Cuervo, 2004; Shintani and Klionsky, 2004; Klionsky, 2005, 2007; Massey et al., 2006; Levine and Kroemer, 2008; Mizushima et al., 2008; Ward et al., 2016). Three major types of autophagy are chaperone-mediated autophagy, micro-autophagy, and macro-autophagy (see Klionsky, 2005; Massey et al., 2006). Whereas, chaperone-mediated autophagy is primarily involved in degrading soluble proteins within the cell, micro-autophagy recruits lysosomes to capture and degrade cytosolic components. Macro-autophagy is a process that first targets, and then engulfs, all or parts of damaged organelles such as mitochondria (mitophagy) or endoplasmic reticulum (reticulophagy). Three major steps in autophagy include; (a) initiation of phagophore membrane formation, (b) elongation of the phagophore, and (c) maturation of the completed autophagosome. Material within the autophagosome are then degraded by various enzymes (e.g., lysozymes) and the components used either as an energy source, recycled (e.g., protein synthesis), or eliminated by exocytosis. An increase in adenosine monophosphate kinase (AMPK) in response to energy demand, detected by an increase in the AMP to ATP ratio, is a strong activator of autophagy (see Ward et al., 2016). An example of this was reported in a study conducted on *C. elegans* mutants that were defective in feeding activity in which autophagy was activated to increase energy production from fat and lipid break down (Mörck and Pilon, 2006). Inhibition of autophagy occurs in response to increased activity of mammalian target of rapamycin (mTOR). An increase in mTOR levels occurs in response to elevations in amino acid levels and is a major signal to enhance protein synthesis (Ward et al., 2016).

Although the autophagy pathway has been characterized in yeast and mammalian cells and animals, it was only recently characterized in avian species (Piekarski et al., 2014). In male and female jungle fowl, which represent a progenitor of commercial egg laying chickens and meat chickens (broilers), it was determined that autophagy-related gene expression was both tissue and gender dependent that concurs with previous reports in mammals (Komatsu et al., 2007; Coto-Montes et al., 2009; Du et al., 2009; Vega-Naredo et al., 2009). In Japanese Quail lines divergently selected for susceptibility or resistance to stress (based on plasma corticosterone levels following a brief period of restraint, see Satterlee and Johnson, 1988), there were also tissue and genotype dependent differences in autophagy related genes representing each of the three steps of autophagosome formation (Piekarski et al., 2014). A phenogram, constructed from nucleotide sequences of the 14 autophagy-related genes that were investigated, exhibited high homology with their mammalian orthologs and were consistent with the consensus view of vertebrate evolution (Piekarski et al., 2014).

Another important component of cells for quality control of proteins is the proteosome which hydrolyzes proteins following ubiquitination (see review Tanaka, 2009). Damaged proteins, for example following oxidation, are first tagged by ubiquitin and then directed to the proteosome. The proteosome is composed of approximately 54 proteins arranged into a 20S core of 14 proteins and two 19S regulatory components (20 subunits) that act as lids for entry of proteins into, and release of amino acids from, the proteosome.

Feed efficiency (FE) remains one of the most important genetic traits in commercial poultry and livestock production since the majority of cost (up to 70%) in raising an animal to market weight is associated with animal feed (Emmerson, 1997; Aggrey et al., 2010; Willems et al., 2013). Commercial broilers have been highly selected for growth and conversion of feed into meat (feed efficiency, FE). Studies conducted in a pedigree male (PedM) broiler line indicate that mitochondria isolated from breast muscle exhibited site-specific defects in electron transport resulting in higher mitochondrial reactive oxygen species (ROS) in mitochondria obtained from animals exhibiting a low FE (LFE) phenotype relative to mitochondria from the HFE phenotype (Bottje et al., 2002). The most consistent finding in several studies was an elevation of protein carbonyls (an indicator of protein oxidation), associated with LFE that was accentuated in the mitochondrial fraction of breast muscle of the LFE phenotype (see Bottje and Carstens, 2009). With evidence of increased oxidative damage it is reasonable to hypothesize that the autophagy and proteosome pathways would be enriched in the LFE PedM broiler phenotype. Thus, this study was conducted to determine the relative expression levels of autophagy-related genes in breast muscle of autophagosome formation between PedM broilers exhibiting HFE and LFE phenotypes. Data mining of global gene and protein expression datasets obtained on the same tissue samples (Kong et al., 2016; Bottje et al., 2017a) was also conducted to assess enrichment of pathways using binomial distribution analysis as previously described (Bottje et al., 2017b,c). The results indicate that rather than being enriched in LFE, autosome and proteosome expression was elevated in breast muscle of the HFE phenotype.

## Materials and methods

### Ethics statement

The present study was conducted in accordance with the recommendations in the guide for the care and use of laboratory animals of the National Institutes of Health. All procedures for animal care were reviewed and approved by the University of Arkansas Institutional Animal Care and Use Committee (IACUC): Protocol #14012.

### Animals-tissues

Breast muscle samples analyzed in the present study were obtained previously (Kong et al., 2011). Briefly, 100 PedM broilers were individually phenotyped for LFE and HFE between 6 and 7 weeks of age. Those with the highest and lowest FE (*n* = 6/group) were selected for gene and protein expression studies. Following cervical dislocation, breast muscle was obtained from 6 birds per group exhibiting a HFE or LFE phenotype. Breast muscle was rapidly excised, flash frozen in liquid nitrogen, stored at −80°C.

### Quantitative real-time polymerase chain reaction (qPCR)

Total RNA was extracted from breast muscle by Trizol reagent (catalog #15596018, Life Technologies) according to manufacturer's recommendations, DNAase treated and reverse transcribed (catalog #95048-100, Quanta Biosciences). The quality and integrity of RNA was assessed using 1% agarose gel electrophoresis and RNA concentrations and purity were determined for each sample by Take 3 micro volume plate using Synergy HT multi-mode microplate reader (BioTek, Winooski, VT). The RT products (cDNAs) were amplified by real-time quantitative PCR (Applied Biosystems 7500 Real-Time PCR system) with Power SYBR green Master Mix (catalog #4312074, Life Technologies). Oligonucleotide primers used for avian autophagy-related gene expression for AMP activated protein kinase α1 (AMPKα1), mechanistic target of rapamycin (mTOR), Beclin 1 (BECN1), and autophagy genes (Atg) (Atg16L1, Atg7, and Atg3) and for the 18S ribosomal housekeeping gene are shown in Table 1. The qPCR cycling conditions were 50°C for 2 min, 95°C for 10 min followed by 40 cycles of a two-step amplification program (95°C for 15 s and 58°C for 1 min). At the end of the amplification, melting curve analysis was applied using the dissociation protocol from the Sequence Detection system to exclude contamination with unspecific PCR products. Relative expressions of target genes were determined by the 2^−ΔΔ*Ct*^ method (Schmittgen and Livak, 2008) and the LFE gene expression was normalized to 1.0 for comparison with the HFE group.

**Table 1:** Oligonucleotide PCR primers.

**Gene**	**Accession No.^a^**	**Primer sequence**	**Orientation**	**Product Size (bp)**
mTOR	XM_417614.5	CATGTCAGGCACTGTGTCTATTCTC	Forward	77
		CTTTCGCCCTTGTTTCTTCACT	Reverse	
AMPKα1	NM_001039603.1	CCACCCCTGTACCGGAAATA	Forward	68
		GGAAGCGAGTGCCAGAGTTC	Reverse	
Beclin1	NM_001006332	TGCATGCCCTTGCTAACAAA	Forward	61
		CCATACGGTACAAGACGGTATCTTT	Reverse	
Atg3	NM_001278070	GAACGTCATCAACACGGTGAA	Forward	65
		TGAGGACGGGAGTGAGGTACTC	Reverse	
Atg7	NM_001030592	ACTGGCAATGCGTGTTTCAG	Forward	66
		CGATGAACCCAAAAGGTCAGA	Reverse	
Atg16L1	XM_003641751	TGCATCCAGCCAAACCTTTC	Forward	65
		CGACGCTGGTGGCTTGTC	Reverse	
18S	AF173612	TCCCCTCCCGTTACTTGGAT	Forward	60
		GCGCTCGTCGGCATGTA	Reverse	

a *Accession number from Genbank (NCBI)*.

### Global expression data

Global protein and gene expression data used in data-mining in the present study were obtained by shotgun proteomics and RNAseq analysis, respectively, conducted on the same breast muscle tissues as part of previous studies (see Kong et al., 2016; Bottje et al., 2017a). Extracted proteins were subjected to shotgun proteomics analysis by in-gel trypsin digestion followed by tandem mass spectrometry at the Proteomics Core Laboratory (University of Arkansas for Medical Sciences, Little Rock, AR). Raw mass spectromic data were analyzed using the Mascot search engine (Matrix Science, Boston MA), the UniProtKB database (https://www.uniprot.org/help/uniprotkb), and the results compiled using the Scaffold program (Proteome Software, Portland, OR).

Extracted RNA from breast muscle samples of HFE and LFE PedM broilers were sent to the Research Support Facility at Michigan State University (East Lansing, MI) for 100 base paired end read sequencing using an Illumina HiSeq. The GLC Genomics Workbench 8 was used to map the reads to *Gallus gallus* genome assembly version 4 as recommended by Mortazavi et al. (2008).

### Statistical analysis

Data were analyzed by Student's *t*-test using the Graph Pad Prism version 6.00 for Windows, Graph Pad Software, La Jolla California USA. Differences were considered significant at *P* ≤ 0.05. Binomial distribution analysis was used to assess differences in the numbers of genes and proteins associated with the autophagy pathway as previously described (Bottje et al., 2017b,c). Briefly, the numbers of molecules in which mean values were numerically higher or lower in breast muscle of the HFE compared to the LFE PedM phenotype were determined and used in the exact binomial distribution analysis test offered in the 2010 version of Microsoft Excel^TM^. There was no gating of terms based on significant or fold difference in expression for a given transcript or protein involved in the bionomial distribution analysis that was conducted.

## Results and discussion

Body weight gain, feed intake, and feed efficiency data for PedM broilers presented in Table 2 (previously reported by Kong et al., 2011). The HFE phenotype attained greater efficiency through higher weight gains while consuming the same amount of feed as the LFE phenotype. These data are similar to previous studies in PedM broilers (Bottje et al., 2002; Ojano-Dirain et al., 2004; Iqbal et al., 2005).

**Table 2:** Body weight gain (BW Gain), feed intake (FI), and feed efficiency (FE, BW Gain/FI) in Pedigree Male (PedM) Broilers exhibiting either a high feed efficiency (HFE) or low feed efficiency (LFE) phenotype^a^.

**Animal**	**BW Gain (g)**	**Feed Intake (FI, g)**	**Feed Efficiency (BW Gain/ FI)**
PedM Broiler HFE	630 + 21*	973 + 31	0.65 + 0.01*
PedM Broiler LFE	462 + 16	999 + 38	0.46 + 0.01

a Mean ± SE of 6 observations and (*) represents P < 0.05.

**Figure 1:**
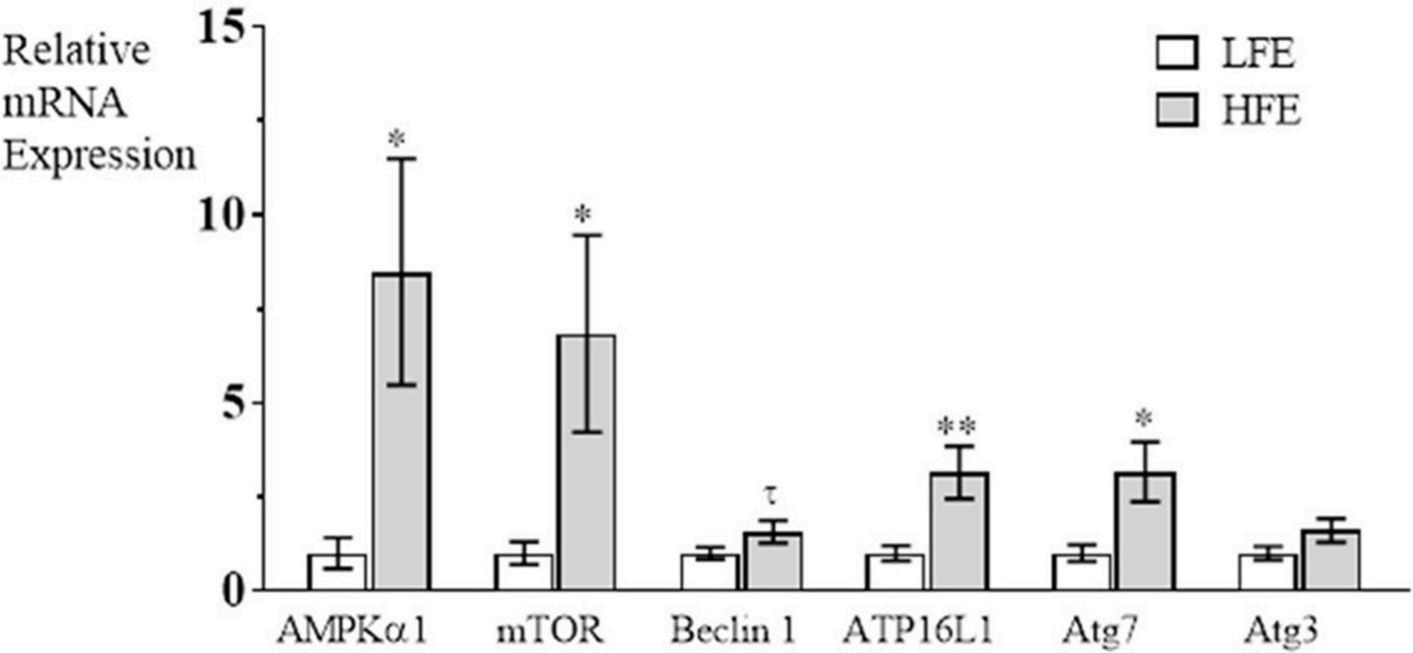
Relative mRNA expression (fold difference) of genes involved in the autophagy pathway in breast muscle of Pedigree Male broilers exhibiting high feed efficiency (HFE) compared to expression in the low feed efficiency (LFE) phenotype. qRT-PCR was used to determine mRNA expression of AMPKα1 (AMP activated protein kinase α1), mTOR (mechanistic target of rapamycin), Beclin1 (Bcl-2 interacting protein), autophagy (Atg) Atg16L1, Atg7, and Atg3. Bars represent mean ± SE (*n* = 6) with ^**^, ^*^, and τ representing *P* < 0.01, *P* < 0.05, and *P* = 0.07, respectively.

The autophagy pathway has been characterized to have three major steps in autophagosome formation; Step 1 (induction of phagosome formation), Step 2, (elongation of phagophore), Step 3 (vacuole closure and autophagosome formation is complete) (Klionsky, 2005). In the present study, it was determined that mRNA expression was elevated in breast muscle in the HFE group for AMPKα1, and mTOR (induction), for Atg16L1 and Atg7 (autophagosome formation), but there were no differences between groups for Beclin 1 or Atg3 expression (Figure 1). The upregulation of both AMPKα1 and mTOR in PedM broilers represent competing signals for activation of the autophagy pathway; mTOR would stimulate protein synthesis and inhibit autophagy initiation whereas elevations in AMPKα1 would stimulate energy production via autophagy, lipolysis and glycolysis, and would inhibit energy consuming pathways (e.g., protein synthesis) (Ward et al., 2016). Since AMPK increases PGC1α expression that stimulates mitochondrial biogenesis and oxidative phosphorylation, it appears that both energy production and protein synthesis, are activated in the HFE phenotype. This appeared to be the case in HFE phenotype Japanese Quail that exhibited higher levels of mTOR mRNA expression, but lower AMPKα1 expression in breast muscle compared to those exhibiting a LFE phenotype (Piekarski, 2015).

**Figure 2:**
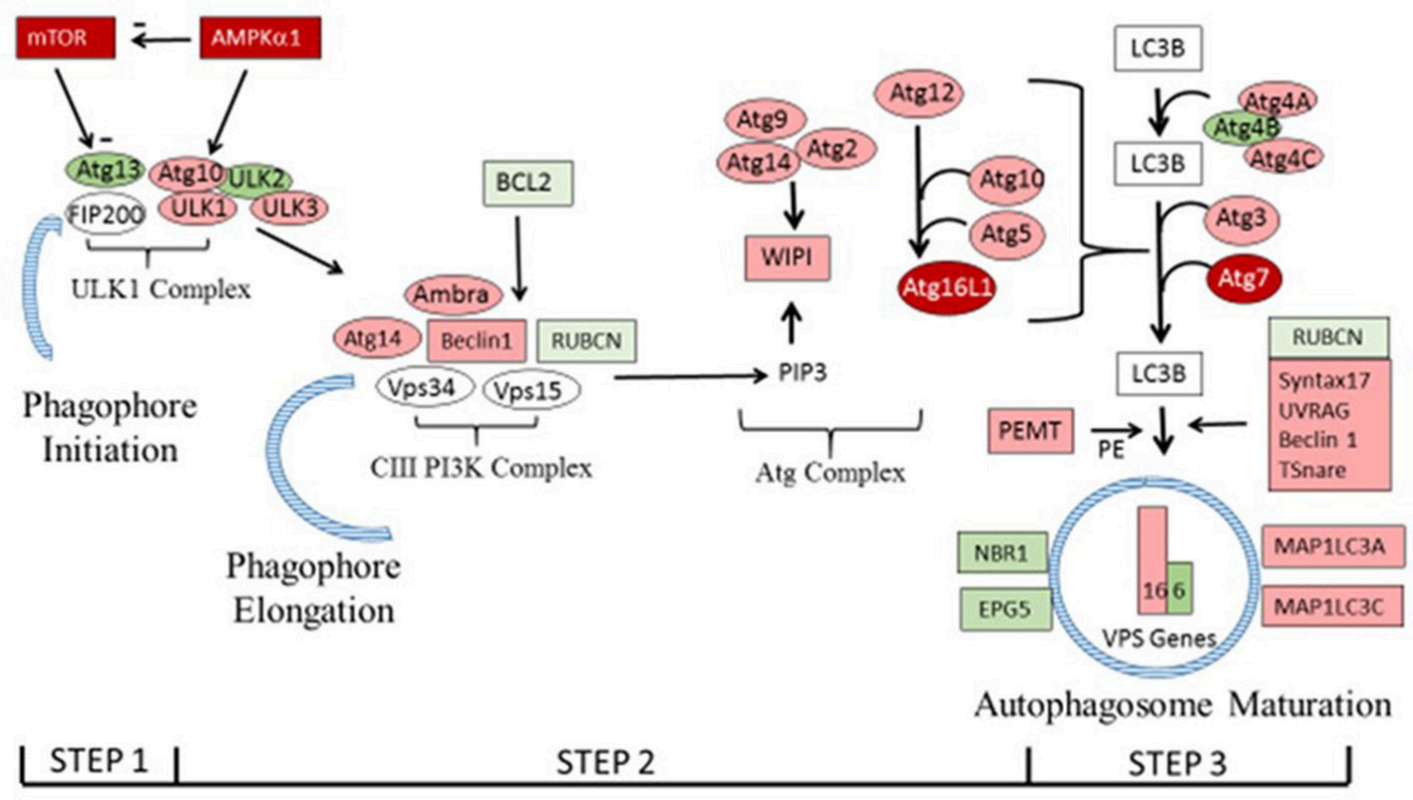
The autophagy pathway showing differential expression of genes in the present study. Genes shown in dark red were differentially expressed as determined by PCR in Figure 1. Genes in pink and green that were higher or lower, respectively, in breast muscle of the HFE Pedigree Male broiler compared to those with LFE as shown in Tables 3, 4 (see text for details). Vacuole sorting protein (VPS) genes are shown as bars for simplicity. The figure was based on one provided in Piekarski et al. (2015) with modifications.

Lists of genes associated with the autophagy pathway and vacuole formation obtained by RNAseq analysis in a previous study (Bottje et al., 2017a) are presented in Tables 3, 4 respectively. In both tables, pink boxes denote genes whose expression level was numerically higher in the HFE muscle and green boxes are genes that were numerically lower in HFE compared to the LFE phenotype. It can be seen that 3 of 5 genes were higher in the HFE phenotype in the initiation step, 7 of 9 were higher in HFE in the second step (isolation—elongation), and 16 of 21 genes were elevated in the HFE phenotype in the final autophagosome formation step (Table 3). Binomial distribution analysis revealed a significant skew of autophagy pathway genes favoring the HFE phenotype (*P* < 0.01). There was also enrichment of genes in the HFE associated with vacuole formation (Table 4); 16 of 22 were numerically higher in the HFE phenotype (Binomial Distribution *P*-value = 0.04). Although these may not be specifically associated with vacuole formation in the autophagy pathway, this enrichment could nonetheless enhance formation of the autophagosome in muscle of the HFE phenotype. Only four proteins were detected that were associated with autophagy or vacuole formation from the proteomics dataset (Kong et al., 2016). All four proteins that were detected were higher in the HFE phenotype (Table 5). A visual representation of the gene expression data in this study is provided in Figure 2 (adapted from Piekarski et al., 2015).

**Table 3:** Autophagy-related gene expression obtained (from RNAseq dataset; Bottje et al., 2017a) in breast muscle of Pedigree Male (PedM) broilers exhibiting high (HFE) and low (LFE) feed efficiency phenotypes.

			**Step**		
**M**	**Gene symbol**	**Gene name**	**1**	**2**	**3**
0.29	ULK1	unc-51 like autophagy activating kinase 1	Y		
0.24	ULK3	unc-51 like kinase 3	Y		
0.17	ATG10	autophagy related 10	Y		Y
−0.04	ATG13	autophagy related 13	X		
−0.51	ULK2	unc-51 like autophagy activating kinase 2	X		
0.18	BECN1	beclin 1		Y	Y
0.17	WIPI1	WD repeat domain, phosphoinositide interacting 1		Y	
0.12	ATG14	autophagy related 14		Y	
0.1	ATG2B	autophagy related 2B		Y	
0.08	PINK1	PTEN induced putative kinase 1		Y	
0.08	WIPI2	WD repeat domain, phosphoinositide interacting 2		Y	
0.03	AMBRA1	autophagy and beclin 1 regulator 1		Y	
−0.02	RUBCN	RUN and cysteine rich domain containing beclin 1 interacting protein		X	X
−0.12	BCL2	BCL2, apoptosis regulator		X	
0.03	ATG5	autophagy related 5			Y
0.39	STX17	syntaxin 17			Y
0.35	ATG4C	autophagy related 4C cysteine peptidase			Y
0.34	PEMT	phosphatidylethanolamine N-methyltransferase			Y
0.28	ATG9A	autophagy related 9A			Y
0.26	TSNARE1	t-SNARE domain containing 1			Y
0.25	ATG16L1	autophagy related 16 like 1			Y
0.21	UVRAG	UV radiation resistance associated			Y
0.18	MAP1LC3C	microtubule associated protein 1 light chain 3 gamma			Y
0.17	ATG12	autophagy related 12			Y
0.16	ATG3	autophagy related 3			Y
0.15	MAP1LC3A	microtubule associated protein 1 light chain 3 alpha			Y
0.09	ATG4A	autophagy related 4A cysteine peptidase			Y
0.09	ATG7	autophagy related 7			Y
−0.06	ATG4B	autophagy related 4B cysteine peptidase			X
−0.15	NBR1	NBR1, autophagy cargo receptor			X
−0.24	EPG5	ectopic P-granules autophagy protein 5 homolog			X
−0.47	DRAM1	DNA damage regulated autophagy modulator 1			X

*Minus (M) represents log_2_ HFE – log_2_ LFE values. A positive value indicates gene expression was higher in the HFE group and a negative value indicates that the expression was lower in the HFE compared to the LFE value. Pink (Y) or green (X) boxes indicate higher or lower values of autophagy gene expression in HFE vs LFE and their association with Step 1 (Initiation), Step 2 (Elongation) and Step 3 (Autophagosome) formation steps of autophagy. Binomial distribution analysis revealed a significant skew (P < 0.01) of autophagy gene expression in breast muscle of the HFE phenotype compared to the LFE PedM broiler phenotype*.

**Table 4:** Vacuole-related gene expression (from RNAseq dataset; Bottje et al., 2017a) in breast muscle of Pedigree Male (PedM) broilers exhibiting high (HFE) and low (LFE) feed efficiency phenotypes.

**M**	**Gene symbol**	**Gene name**
0.59	VPS37C	VPS37C, ESCRT-I subunit
0.56	VPS72	vacuolar protein sorting 72 homolog
0.39	VPS37A	VPS37A, ESCRT-I subunit
0.39	VPS51	VPS51, GARP complex subunit
0.20	VPS26B	VPS26, retromer complex component B
0.19	VPS18	VPS18, CORVET/HOPS core subunit
0.19	VPS33B	VPS33B, late endosome and lysosome associated
0.18	VPS53	VPS53, GARP complex subunit
0.14	VPS45	vacuolar protein sorting 45 homolog
0.13	VPS54	VPS54, GARP complex subunit
0.11	VPS37B	VPS37B, ESCRT-I subunit
0.05	VPS13A	vacuolar protein sorting 13 homolog A
0.04	VPS41	VPS41, HOPS complex subunit
0.04	VPS4B	vacuolar protein sorting 4 homolog B
0.03	VPS36	vacuolar protein sorting 36 homolog
0.01	VPS26A	VPS26, retromer complex component A
−0.05	VPS13C	vacuolar protein sorting 13 homolog C
−0.08	VPS35	VPS35 retromer complex component
−0.14	VPS29	VPS29, retromer complex component
−0.17	VPS13B	vacuolar protein sorting 13 homolog B
−0.22	VPS13D	vacuolar protein sorting 13 homolog D
−0.25	VPS39	VPS39, HOPS complex subunit

*Minus (M) represents log_2_ HFE – log_2_ LFE values. A positive value indicates gene expression was higher in the HFE group and a negative value indicates that the expression was lower in the HFE group compared to the LFE value. Pink or green boxes indicate higher or lower values of vacuole formation in the HFE PedM broiler phenotype. The binomial distribution analysis revealed a significant skew (P = 0.04) of vacuole gene expression in breast muscle of the HFE phenotype compared to the LFE PedM broiler phenotype*.

**Table 5:** Vacuole-related protein expression (obtained from Kong et al., 2016) in breast muscle of Pedigree Male (PedM) broilers exhibiting high (HFE) and low (LFE) feed efficiency phenotypes.

**Fold diff**	**Protein symbol**	**Protein name**
1.71	VPS29	Vacuolar protein sorting-associated protein 29 (Fragment)
6.00	VPS13A	Vacuolar sorting-associated protein 13A (uncharacterized)
1.75	VPS26A	Vacuolar sorting associated protein 26A (uncharacterized)
1.29	VPS35	Vacuolar protein sorting-associated protein 35

*The values are presented as fold difference in expression between HFE and LFE groups. Pink boxes indicate that expression was up-regulated in the HFE compared to LFE phenotype. The binomial distribution analysis P-value = 0.065*.

**Figure 3:**
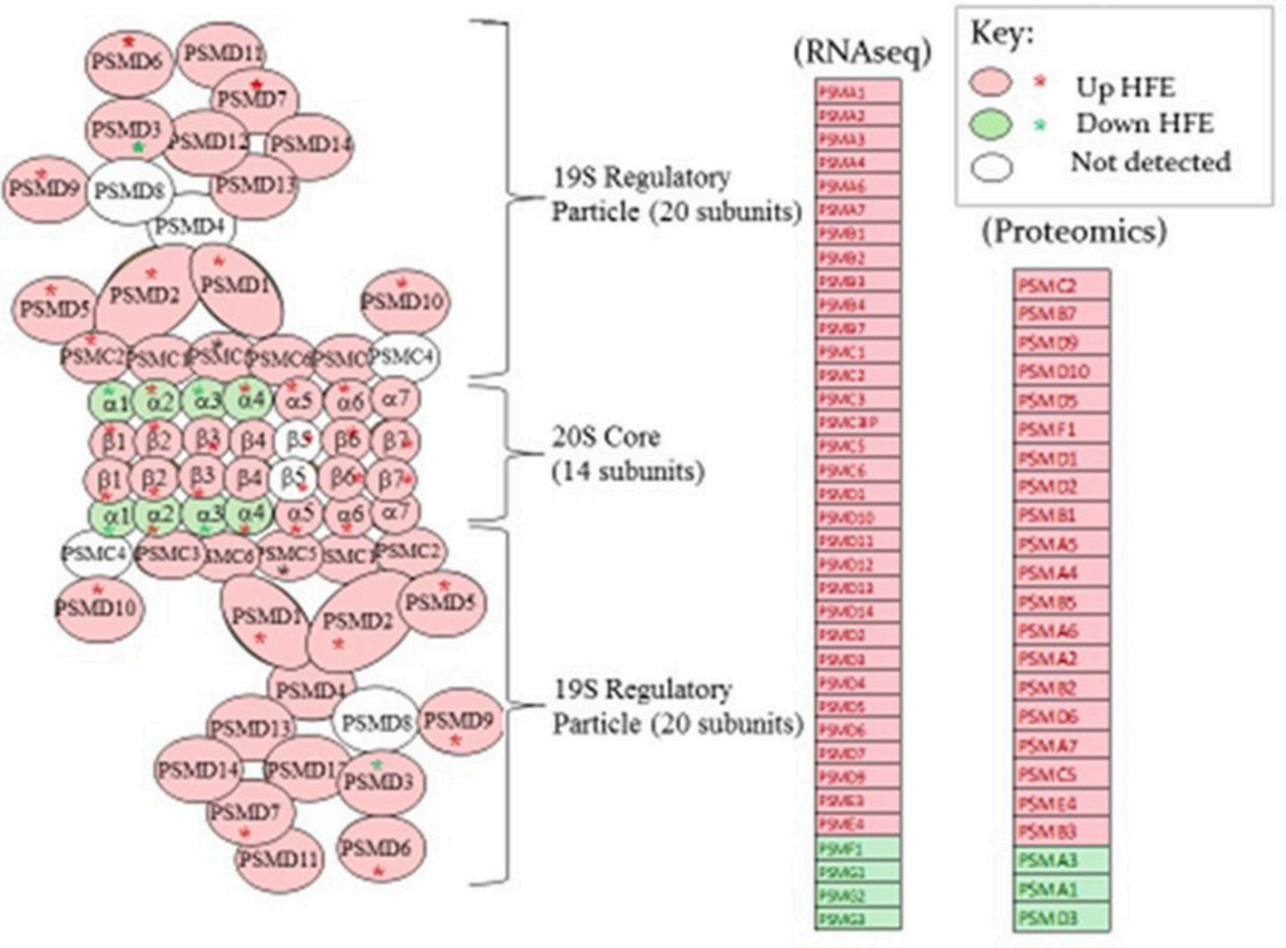
A proteosome subunit schematic from Tanaka (2009) (left side of figure) is shown with expression of genes determined by RNAseq and proteins by proteomics shown in the list to the right in breast muscle that were higher (pink fill) or lower (green fill) in the HFE Pedigree Male (PedM) compared to the LFE phenotype. Open ovals indicate that the gene was not detected in the RNAseq dataset. Proteomic data is projected on the schematic from the list using red or green asterisks. Bionomial distribution analysis for the RNAseq and proteomic data were *P* = 0.00001 and *P* = 0.0002, respectively.

A list of genes associated with proteosome subunits detected in the RNAseq dataset is provided in Table 6. A highly significant skew favoring expression of proteosome-related genes in the HFE PedM broiler was detected (binomial *P*-value = 0.000001). Projection of this gene expression data onto a schematic of the 26S proteosome by Tanaka, 2009s provided in Figure 3. In the shotgun proteomic dataset reported by Kong et al. (2016), there was also a significant skew in expression of proteosome proteins favoring the HFE phenotype (binomial *P*-value = 0.0002). These data would indicate that proteosome expression would be enhanced in the HFE phenotype.

**Table 6:** List of genes obtained from RNAseq dataset (from Bottje et al., 2017a) involved in proteosome formation in breast muscle of Pedigree Male (PedM) broilers exhibiting high (HFE) and low (LFE) feed efficiency phenotypes.

**M**	**Gene symbol**	**Gene name**
0.60	PSMA1	proteasome subunit alpha 1
0.34	PSMA2	proteasome subunit alpha 2
0.32	PSMA3	proteasome subunit alpha 3
0.31	PSMA4	proteasome subunit alpha 4
	PSMA5	proteosome subunit alpha 5
0.30	PSMA6	proteasome subunit alpha 6
0.28	PSMA7	proteasome subunit alpha 7
0.23	PSMB1	proteasome subunit beta 1
0.23	PSMB2	proteasome subunit beta 2
0.22	PSMB3	proteasome subunit beta 3
0.21	PSMB4	proteasome subunit beta 4
	PSMB5	proteosome subunit beta 5
	PSMB6	proteosome subunit beta 6
0.21	PSMB7	proteasome subunit beta 7
0.21	PSMC1	proteasome 26S subunit, ATPase 1
0.20	PSMC2	proteasome 26S subunit, ATPase 2
0.19	PSMC3	proteasome 26S subunit, ATPase 3
0.18	PSMC3IP	PSMC3 interacting protein
0.17	PSMC5	proteasome 26S subunit, ATPase 5
0.15	PSMC6	proteasome 26S subunit, ATPase 6
0.15	PSMD1	proteasome 26S subunit, non-ATPase 1
0.15	PSMD10	proteasome 26S subunit, non-ATPase 10
0.13	PSMD11	proteasome 26S subunit, non-ATPase 11
0.10	PSMD12	proteasome 26S subunit, non-ATPase 12
0.09	PSMD13	proteasome 26S subunit, non-ATPase 13
0.08	PSMD14	proteasome 26S subunit, non-ATPase 14
0.06	PSMD2	proteasome 26S subunit, non-ATPase 2
0.06	PSMD3	proteasome 26S subunit, non-ATPase 3
0.05	PSMD4	proteasome 26S subunit, non-ATPase 4
0.03	PSMD5	proteasome 26S subunit, non-ATPase 5
0.02	PSMD6	proteasome 26S subunit, non-ATPase 6
0.02	PSMD7	proteasome 26S subunit, non-ATPase 7
0.02	PSMD9	proteasome 26S subunit, non-ATPase 9
0.02	PSME3	proteasome activator subunit 3
0.01	PSME4	proteasome activator subunit 4
−0.03	PSMF1	proteasome inhibitor subunit 1
−0.04	PSMG1	proteasome assembly chaperone 1
−0.04	PSMG2	proteasome assembly chaperone 2
−0.11	PSMG3	proteasome assembly chaperone 3

*Minus (M) represents log_2_ HFE – log_2_ LFE values. A positive value (displayed in pink) indicates gene expression was higher in HFE and a negative value (displayed in green) indicates that the expression was lower in the HFE compared to the LFE value. Pink or green indicate higher or lower values of gene expression in the HFE phenotype*.

Thus, the HFE phenotype exhibited enrichment of genes and proteins associated with the autophagy and proteosome pathways which was opposite to what we had originally hypothesized since there was pervasive protein oxidation present in several tissues including muscle in the LFE phenotype (Bottje and Carstens, 2009). Proteosome enrichment indicates that protein degradation and/or quality control may be enhanced in the HFE phenotype. Although protein degradation and resynthesis would represent an energetically expensive process, the HFE phenotype also apparently has the infrastructure to support enhanced capability for energy production and shuttling of ADP and ATP as well as replenishing phosphocreatine in the cytosol (Bottje et al., 2017b) that would be able to support the high amounts of ATP needed for protein synthesis. In addition, part of the benefit of protein degradation and resynthesis may come by maintaining optimal activity of the proteins in carrying out their specific functions. For example, the proteosome may facilitate functionality by maintaining the tertiary structure when proteins become misaligned through refolding activity (Ciechanover, 1998; Hershko and Ciehanover, 1998).

In summary, both the autophagy and proteosome pathways were enriched in breast muscle of the HFE PedM broiler. We do not know if the enrichment of autophagy pathway genes downstream from mTOR and AMPKα1 was due to inherent differences or signal transduction mechanisms. We also do not know if enrichment of both the autophagy and proteosome expression in breast muscle in the HFE phenotype is sufficient to enhance protein quality and activity. However, if these processes are enhanced in the HFE phenotype, this would provide a means to maintain higher structural integrity and activity of proteins as well as organelles (e.g., mitochondria, endoplasmic reticulum) through continual removal of damaged components within the cell.

## Author contributions

AP-W, BK, SD, and WB designed and/or conducted the studies. The analysis of data was carried about by the same authors with EG and KL. The paper was written through contributions and critical review by all authors.

### Conflict of interest statement

AP-W is employed by Adisseo USA, Alpharetta Georgia. The remaining authors declare that the research was conducted in the absence of any commercial or financial relationships that could be construed as a potential conflict of interest. The reviewer DC and handling editor declared their shared affiliation at the time of the review.
